# Aspirin and statin therapy in sepsis, a red herring?

**DOI:** 10.1186/2197-425X-3-S1-A227

**Published:** 2015-10-01

**Authors:** R Campbell, A McGuire, L Young, A Mackay

**Affiliations:** Victoria Infirmary/ NHS Greater Glasgow & Clyde, Glasgow, United Kingdom

## Intr

Severe sepsis is attributable to 37000 deaths per year in the United Kingdom [1]. Recent studies have suggested aspirin or statins have a beneficial effect in the treatment of sepsis, reducing overall mortality [2–4]. This is thought to be secondary to their anti-inflammatory effect, blocking inflammatory cascades triggered in sepsis [2, 3].

## Objectives

To identify whether aspirin therapy, statin therapy or combination therapy improves survival in septic patients admitted to ICU compared to patients receiving neither.

## Methods

Patients admitted with sepsis to a 5 bedded teaching hospital intensive care unit over a 2 year period from January 2013 to March 2015 were identified utilising the WardWatcher™ database. Demographic data, outcome data and medications prescribed during this time were obtained via the ICU clinical information system, specifically looking for aspirin and statin therapy prior to admission. This information was subsequently analysed using an Excel™ spreadsheet.

## Results

During the study period, there were a total of 218 (35.7%) admissions to ICU identified with sepsis out of a total of 611 admissions. Ten were excluded due to incomplete data. Septic patients were split into four groups; aspirin therapy, dual aspirin and statin therapy, statin therapy, neither aspirin nor statin therapy.

The majority (61%) of patients admitted with sepsis were on neither aspirin nor statin therapy. This patient group also had the lowest median age of 56. Median APACHE-II scores were similar in each group and this was reflected in the lack of any significant survival difference between groups. Patients on neither therapy were more likely to survive hospital admission (67.7%) than those on dual (57.6%) or single aspirin or statin therapy, although this was not statistically significant.

## Conclusions

Our study demonstrates no significant benefit in outcome from sepsis in intensive care with aspirin or statin therapy.

**Table 1:** Medications prescribed

	Aspirin therapy	Statin therapy	Dual therapy	Neither therapy
Total number	12	36	33	127
Male:Female ratio	1:1	1:1.25	1:1.2	1:1.1
Median age (IQR)	70.5 [61.25-78.25]	66 [52.25-75]	72 [63-77]	56 [47-68.5]
Surgical:Medical	1:3	1:2	1:0.7	1:0.69
Median APACHE-II (IQR)	17.5 [15.5-24.5]	20 [17-28]	20 [16-26]	20 [14-27]
% Hospital survival	66.7	63.9	57.6	67.7
